# Correction: Psychometric properties of the Bangla version of the sense of coherence scale among university students in Bangladesh

**DOI:** 10.1371/journal.pone.0328725

**Published:** 2025-07-18

**Authors:** Momtaz Sultana, Yuta Hayashi, Tanzilur Rahman Tamim, Rie Chiba, Muhammad Kamal Uddin

## Notice of Republication

An incorrect version of S1 File was published in error. This article was republished on June 30, 2025 to correct this error. Please download this article again to view the correct version.
