# Supplementary material for: Psychometric properties of the Bangla version of the sense of coherence scale among university students in Bangladesh
Source: PLoS One. 2025 Apr 1;20(4):e0320896. doi: 10.1371/journal.pone.0320896 (PMC11960917; doi:10.1371/journal.pone.0320896)
Supplement: Attachment — Submitted filename: Response_to_Reviewers_auresp_2.docx [file pone.0320896.s007.docx]

**RESPONSE TO REVIEWERS**

Dear Editor and Reviewers,

We sincerely appreciate the time and effort you have dedicated to reviewing our manuscript. We are deeply grateful to the editor and reviewers for your insightful comments and valuable feedback, which have greatly contributed to improving the quality of our work.

We have revised the manuscript in accordance with the review comments. Our responses are presented in detail as follows, as well as a revised version of the manuscript with track changes. We have also reviewed our reference list and removed Ilyas’s literature because it was found that the original text is currently unable to be read. The removal of this paper will not change the content of our text (Line 166-167).

We have re-read the revised manuscript whether the article's language adhere to the journal's standards. If there are any obvious linguistic or grammatical errors, please let us know.

**Response to Comments from Reviewer #2**

**Comment #2-1:**

*Line 106- 108; “Therefore, this study aimed to translate the original English SOC-13 into Bangla and examine its construct validity and reliability based on its internal consistency among Bangladeshi university students*

*Comment: The sentence above implies that both construct validity and reliability were assessed using internal consistency. To improve clarity, the authors should rephrase this. Additionally, since the latter part of the manuscript specifies that both structural and convergent validity were tested, it would be more accurate to state that "validity and reliability" were assessed at this stage. Now that the methods section includes detailed descriptions of the types of validity measurements conducted, this will provide clearer information for the readers.*

**Response:**

Thank you for your insightful comment. We have revised the sentence to improve clarity (Line 109-110): “*Therefore, this study aimed to translate the original English SOC-13 into Bangla and examine its validity and reliability among Bangladeshi university students.*”

****************************

**Comment #2-2:**

*Line 138-140; The authors' sample size calculation and their decision to include a larger sample size are not aligned. It is rather speculative than scientifically convincing. Both smaller and larger sample sizes than calculated have limitations regarding methodological rigor and ethical considerations.*

**Response:**

Thank you for providing helpful feedback on our sample size estimate and decision to incorporate a larger sample. We would like to clarify our approach. Based on the COSMIN guidelines, we determined that the minimum sample size for the SOC-13 scale is 130 cases, or 10 case each item. This figure ensured that we met the fundamental prerequisites for performing confirmatory factor analysis (CFA). However, we chose a larger sample size to strengthen our results, addressing any missing data. Considering larger sample sizes are commonly used in psychometric research to obtain robust results, we hope it is still acceptable.

****************************

**Comment #2-3:**

*Line 207-208; convergent validity was examined using correlations with self-esteem, well-being, and psychological distress
Comment: Isn’t it discriminant validity that the authors wish to test between SOC and psychological distress?*

**Response:**

Thank you very much for your insightful comment. We apologize for the insufficient response to your previous comments (#2-1 and #2-13), despite having received similar feedback before. As you pointed out, sense of coherence and psychological distress are distinct concepts, and thus it is not appropriate to examine convergent validity based on their relationship. Our intention in employing K6 for assessing psychological distress in this study was to investigate the criterion validity of the Bangla SOC-13 because previous studies reported a high negative correlation between them. To present this, we revised the manuscript as follows (Line 209-211): “...*convergent and criterion validity were examined using correlations with self-esteem and well-being, and with psychological distress, respectively.*” We have also revised the relevant description in the manuscript in line with this modification (Line 41-43, Line 269, Line 321).

****************************

**Comment #2-4:**

*Line 329-330- ‘Second, there was a lack of assessment of the items and cross-cultural validity’.
I assume that authors meant to say that items and concept of the questionnaire was not tested for its’ conceptual validity. Would suggest the authors to rephrase according to their intention.*

**Response:**

Thank you for the feedback. We revised the article to address the issues of conceptual validity and cross-cultural applicability. We have added the following line to the limitations section (Line 329-330): “*Second, cross-cultural applicability was not rigorously examined.*”

****************************

**Comment #2-5:**

*Line 340-341- what about the convergent and discriminant validity? The study has shown significant positive correlation between SOC and similar construct as well as significant negative correlations between SOC and dissimilar construct. Perhaps the authors can state that in the conclusion.*

**Response:**

Based on the results regarding the validity, we have added the following description to the conclusion section (Lines 340-343): “*The scale demonstrated good construct validity, including acceptable structural and convergent validity; acceptable criterion validity; good overall reliability based on internal consistency.*" We have also revised the relevant description in the abstract (Line 47-48).

****************************

**Comment #2-6:**

*Authors should discuss the study's implications in the conclusion section, considering its originality.*

**Response:**

Thank you for your comment. We have described the study's implications as follows (Lines 344-347): “*Despite these limitations, the findings have significant implications for psychology and public health research in Bangladesh. The validated Bangla SOC-13 can be a useful instrument for future SOC studies and strategies to enhance well-being.*"
