# Supplementary material for: Psychometric properties of the Bangla version of the sense of coherence scale among university students in Bangladesh
Source: PLoS One. 2025 Apr 1;20(4):e0320896. doi: 10.1371/journal.pone.0320896 (PMC11960917; doi:10.1371/journal.pone.0320896)
Supplement: Attachment — Submitted filename: Response to Reviewers.docx [file pone.0320896.s004.docx]

**Response to the Comments in the decision letter**

Dear person in charge of the editorial office,

We appreciate the time and effort by the editor in reviewing this manuscript and allowing us to resubmit it for publication in *PLOS ONE*. We have revised our manuscript in accordance with the comments in the decision letter. Our responses are presented in detail as follows. We trust that the revised manuscript will be found suitable for publication.

**Comment**

*We recognize that you obtained ethical approval from your local ethics committee after you began your study: your ethics approval document is dated 13 July 2021, while you began collecting patient samples from 01 July to 31 Oct 2021. You also state that informed consent was obtained from all participants, which indicates that the samples were prospectively collected for research purposes. For human subjects research, PLOS ONE requires that authors obtain ethics committee approval before beginning the study, and we do not accept retrospective ethical approval.*

**Response**

We apologize for the incorrect description regarding the survey period. The correct period was "29th July to 31st October, 2021." We have checked the raw data and confirmed all of our research data were collected after obtaining permission from the Ethics Review Board. The data collection dates were recorded in the columns "Start Date (column 3)" and "End Date (column 4)" on pages 1-5 in the file named “Raw data”.
