# Supplementary material for: Psychometric properties of the Bangla version of the sense of coherence scale among university students in Bangladesh
Source: PLoS One. 2025 Apr 1;20(4):e0320896. doi: 10.1371/journal.pone.0320896 (PMC11960917; doi:10.1371/journal.pone.0320896)
Supplement: Attachment — Submitted filename: Response_to_Reviewers_auresp_1.docx [file pone.0320896.s006.docx]

**RESPONSE TO REVIEWERS**

Dear Editor and Reviewers,

We apologize for the delay in submitting the revised manuscript. We appreciate the time and effort by the editor and reviewers in considering this manuscript and allowing us to submit a revised version to PLOS ONE. We are grateful to the editor and reviewers for the constructive feedback and helpful suggestions.

We have revised the manuscript in accordance with the review comments. Our responses are presented in detail as follows, as well as a revised version of the manuscript with changes highlighted in red.

Taking into account the contributions to this study, including the revisions, the order of authors has been rearranged. All authors have agreed to this change.

**Response to Comments from Reviewer #1**

**Comment #1-1:***(Introduction) Please provide a detailed review of the relationship between the three variables used as indicators of construct validity (self-esteem, well-being, and psychological distress) and SOC, and then state your hypothesis. For example, what kind of relationships have been shown in previous studies? This information is necessary to determine whether each indicator is appropriate as an indicator of construct validity.*

**Response:**

Thank you for your valuable comments. The relationship between the three variables has been added with references (lines 74-80) as follows: “*People with high self-esteem are more likely to employ GRRs that reinforce SOC. Strong SOC correlates with positive mental health and well-being and functions as a robust predictor of health-promoting activities. Conversely, an inverse relationship was observed between the SOC and psychological distress. In clinical populations, lower SOC scores have been linked to higher levels of psychological distress, suggesting that SOC may shield against mental health problems”.*

****************************

**Comment #1-2:***(Instruments) Please use a consistent description for [Instruments].*

*Please indicate the internal consistency coefficient for K6 and WHO-5, and for K6, explain what higher scores mean. Also, please include demographic information (what was listened to and how).*

**Response:**

Thank you for the comments. We added the mentioned descriptions of the instruments. Please check in the instruments section.

****************************

**Comment #1-3:***(Results) Please provide descriptive statistics for each measure.*

*In SOC-13, correlations between each factor are reported as [internal consistency reliability], which I do not think is suitable as an indicator of reliability. In CFA, correlations between each factor are calculated, which can cope with the issue of attenuation of correlation coefficients (i.e., values close to the true correlation coefficient are calculated). I think it would be better to report the correlation coefficients between factors based on the CFA results in the chapter [Factorial validity]. Please consider this.*

**Response:**

The correlation coefficients between factors based on CFA results has been added in the structural validity section (lines: 252-254).

****************************

**Comment #1-4:** *Translation process of the SOC-13 scale (p.5, l.101~)*

*MS, TRT, and MKU refer to authors? It is unclear what they stand for, so please add a note or state "first author" etc.*

**Response:**

We apologize for the confusing description. We have revised it to state that “first author” etc., respectively (lines 118-121).

****************************

**Comment #1-5:** *Sample description and Table1*

*Please make sure the statistics in Table 1 are consistent with those in the text (gender percentage). The total number of students in the grade is 319. Is there one unknown? If that's so, please add it.*

**Response:**

Thank you for noticing it. We revised according to the suggestion (one participant didn’t respond).

****************************

**Comment #1-6:** Figure1

*Please provide an explanation for the numbers and arrows in Figure 1.*

**Response:**

Thanks for the valuable comments. Explanation was added (lines 247-252) as follows: *“Model 2 (Fig1) revealed standardized item loadings across three latent constructs: comprehensibility, meaningfulness, and manageability. This model indicated significant item loadings across most components, although items 1 (0.06), 2 (0.23), and 3 (0.40) had weak loadings in the dimensions of meaningfulness, comprehensibility, and manageability, respectively indicating a poor fit for these items”*.

****************************

**Response to Comments from Reviewer #2**

**Comment #2-1:** *The authors should use consistent terminology throughout the manuscript. Factor analysis is a method for establishing construct validity, while testing against similar and dissimilar constructs is also a method of establishing construct validity. In some literature, this is referred to as testing criterion validity. It appears that the authors have assessed convergent and discriminant validity by examining correlations with other constructs, such as self-esteem, psychological distress, and mental well-being. One could also argue that criterion validity has been established. Therefore, it would be best for the authors to clarify their intentions and ensure the readers have a clear understanding. Please revise the methods section in the abstract, the aim in the introduction (page 5, lines 89–90), the data analysis in the methods section (page 9, line 182), and the relevant sections in the discussion and conclusion accordingly.*

**Response:**

We appreciate your comments. We revised the mentioned sections according to the COSMIN guideline. Please check the abstract (lines 31-33), introduction (lines107-108), and data analysis (lines 207-210) sections. Furthermore, although the analysis could be interpreted as testing criterion validity, our primary aim was to assess construct validity, focusing specifically on structural and convergent validity. We have clarified this in the revised methods section to ensure precise terminology and avoid ambiguity for the readers.

****************************

**Comment #2-2:** *Please include keywords in the abstract that use Medical Subject Headings (MeSH) to facilitate indexing and searching in MEDLINE/PubMed and other databases.*

**Response:**

Thank you for the helpful suggestion. Keywords in the abstract using Medical Subject Headings was added (Bangladesh; Psychometrics; Scale; Sense of coherence; University students).

****************************

**Comment #2-3:** *The authors should address grammatical errors and improve the English writing to enhance readability and comprehension.*

**Response:**

The revised manuscript has been proofread again to avoid grammatical errors and enhance readability.

****************************

Introduction:

**Comment #2-4:** *Lines 55-56: The health model of salutogenesis involves a complex interplay between two key concepts: Sense of Coherence (SOC) and Generalized Resistance Resources (GRRs). The authors should provide readers with information about SOC, including its interaction with GRRs.*

**Response:**

We revised the introduction section based on the reviewer’s helpful suggestion. The interaction between SOC and GRRs as core concepts of salutogenesis has been added in the introduction section (lines 55-64) as follows: *“As significant personal resources in terms of health and well-being, generalized resistance resources (GRRs) and sense of coherence (SOC) are the core parts of salutogenesis. Salutogenesis describes how humans deal with various stressors daily. The term "generalized resistance resources" was coined by Antonovsky in 1979 and 1987 to describe the resources accessible to people, groups, or communities that help them cope with stressors and develop SOC. A person can successfully manage stress using GRRs, which prevent tension induced by stressors from transforming into stress. Through the accumulation of such experiences, an individual's SOC is formed and developed. Strong SOC enables individuals to effectively identify and apply their GRRs in response to stressors”.*

****************************

**Comment #2-5:** *Lines 58-59: SOC is defined as the ability to appropriately utilize GRRs to promote one's own health. The statement in the mentioned lines should be rephrased for better clarity.*

**Response:**

We have rephrased the sentences (lines 63-64) as follows*: “Strong SOC enables individuals to effectively identify and apply their GRRs in response to stressors”.*

****************************

**Comment #2-6:** *Lines 62-66: Please break down this sentence into shorter, more meaningful sentences to improve readability.*

**Response:**

Thanks for the suggestion. We revise the sentences as per the comments (lines 66-70).

****************************

**Comment #2-7:** *Line 72: The statement could be rephrased as, "SOC-13 has been translated and cross-culturally adapted." As noted in the literature, the SOC scale has been tested for its validity and reliability in various contexts, not merely translated.*

**Response:**

We rephrased the sentence (lines 82-83), *“SOC-13 has been translated and cross-culturally adapted”.*

****************************

**Comment #2-8:** *Lines 76-88: The third paragraph of the introduction should be rephrased and realigned. Clearly justify how the health model of salutogenesis and a tool to measure SOC can be utilized to promote mental health and support efforts to mitigate the current mental disease burden in Bangladesh.*

**Response:**

This Paragraph was rephrased and realigned according to the comment (lines 90-105).

****************************

**Comment #2-9:** *Lines 77-79: The authors mention limitations in human resources for mental health and health services separately. These two are undoubtedly interrelated, so please rephrase for better clarity.*

**Response:**

We have rephrased this sentence (lines 91-93) as, *“Despite the high prevalence and significant burden of mental illness, mental health services in Bangladesh are limited”.*

****************************

**Comment #2-10:** *Line 89: It would be helpful if the authors justified in the second and third paragraphs of the introduction why they decided to adapt the shorter version of SOC-13 rather than the original SOC-29.*

**Response:**

Thank you for the valuable suggestion. We have added the justification for using the shorter version of SOC scale (lines 82-87) as follows: *“The SOC-13 is a concise and easier-to-administer version of the SOC-29, resulting in higher response rates and better data quality. It retains good psychometric qualities, with reliability and validity comparable to the SOC-29 and has been demonstrated to be successful in a variety of settings”.*

****************************

Methods:

**Comment #2-11:** *Line 104: Did the authors mean to say "back-translated into English"? Please rephrase for clarity.*

**Response:**

We have rephrased the sentence for clarity (line 121).

****************************

**Comment #2-12:** *Line 116: Please provide information about the sample size. How was the sample size determined, and what sampling method was used?*

**Response:**

We appreciate your comments. Information about the sample was added in the participants and procedures of methods section (lines 134, 137-140). We used convenience sampling technique for data collection and for sample size determination we followed COSMIN guidelines.

****************************

**Comment #2-13:** *Line 182: It appears that the authors have tested for convergent and discriminant validity. Did you use a multitrait-multimethod matrix for this analysis? The analysis could also be interpreted as testing for criterion validity. The authors should use precise terminology and provide detailed descriptions of the analysis methods to avoid any ambiguity for the readers.*

**Response:**

We did not employ a multitrait-multimethod matrix in this study, as our analysis was based on a single method of measurement (Pearson’s correlation coefficients) and aimed to test relationships between SOC-13 and other constructs. We followed the COSMIN guidelines, which support correlation analysis as an appropriate method for construct validity when using a single method.

****************************

Results:

**Comment #2-14:** *Line 200: In Table 1, it appears the row corresponding to "Females" is missing. Please ensure this is included.*

**Response:**

Thank you for mentioning it. We have added the row corresponding to "Female" in Table 1.

****************************

**Comment #2-15:** Line 203: *Ensure consistent terminology throughout the manuscript. Ensure that the most appropriate term is used.*

**Response:**

We ensured that the key terms were used consistently across all sections, including the introduction, methods, results, and discussion. Specifically: The term "structural validity" has been consistently used instead of the previously interchangeable term "factorial validity". We have removed any instances of ambiguity or inconsistency in terminology related to construct validity to ensure clarity.

****************************

**Comment #2-16:** *Table 2: It is unclear what the authors aim to convey by reporting item means and standard deviations. While it is statistically valid to report these, what do they signify in the context of the study? According to Antonovsky’s original concept, SOC is considered a single construct, and he was critical of reporting scores under three different domains due to his belief in its unified nature. Although modern statistical techniques have led to such analyses in the literature, interpreting single-item statistics may not provide meaningful insights for this construct. Therefore, this Table may be redundant. The authors may consider to replace it with a Table that includes factor loadings for each item, which would be more informative. However, authors may accept or decline this suggestion based on valid reasons.*

**Response:**

Table 2 presents the mean values and standard deviations for each item to see whether any item exhibited a ceiling or floor effect. On the other hand, it is incongruous to arrange the items by domain before presenting the results of the factor analysis, so the items were sorted by item number.

****************************

**Comment #2-17:** *Table 3: It would be best to add footnotes indicating the cut-off levels you considered for a good fit. For example, "CFI – Comparative Fit Index (desired >0.9)."*

**Response:**

Thank you for the suggestion. Footnote was added according to the comment (lines 260-263) as follows: *“df, degrees of freedom; GFI, goodness-of-fit index; CFI, comparative fit index (desired ≥ 0.90); TLI, Tucker-Lewis index (desired ≥ 0.90); RMSEA, root mean square error of approximation (desired < 0.08); SRMR, standardized root mean square residual (desired < 0.10)”.*

****************************

**Comment #2-18:** *Line 241: While "test-retest reliability" is a commonly used term, "internal consistency reliability" might not be as widely recognized. Consider revising the sub-title to "Reliability of the Bangla Version of SOC-13" and simply provide the relevant values. Additionally, in the methods section (lines 184-185), clearly state that reliability was measured through internal consistency.*

**Response:**

Thank you for the reviewer’s suggestion. We have revised the subheading to "Reliability of the Bangla Version of SOC-13" (line 273) to use a more widely recognized term. Additionally, we clarified in the methods section (lines 207-210) that reliability was measured through internal consistency, using Cronbach’s alpha as recommended by the COSMIN guidelines.

****************************

Discussion:

**Comment #2-19:** *In general, the discussion should be strengthened to provide a meaningful analysis based on the current study's findings. Therefore, I suggest the authors rewrite the discussion section using the following sub-themes:*

*• Summary of principal findings: Provide a concise summary without reiterating the results.*

*• Strengths and weaknesses of the study: Discuss the key strengths and any limitations encountered during the study.*

*• Comparison with existing evidence: Explain how the current study's findings compare or contrast with the existing literature. This should include evidence that both supports and contradicts the study's findings. When discussing similarities or differences with previous research, base the arguments on the strengths and weaknesses of the methodologies and contexts of the studies rather than personal opinions.*

*• Implications for future research: Discuss the potential impact of the findings on future research directions.*

**Response:**

Thank you for your insightful suggestions regarding the discussion section. We have revised this section to enhance the depth and meaningful analysis of the findings, as per your recommendations.

- We have included a concise summary of the key findings from our study without reiterating the detailed results. This summary now highlights the core outcomes that align with the objectives of the study.
- We have elaborated on the main strengths of our study, while also discussing the limitations we encountered. The discussion now explicitly addresses how these factors could have influenced the results and what future research could do to mitigate these limitations.
- We have expanded the comparison between our findings and existing literature. This includes discussing studies that both support and contrast with our results.
- We have discussed the potential implications of our findings for future research.

We believe these revisions strengthen the discussion and provide a more meaningful analysis of the study’s findings.

****************************

Specific Comments:

**Comment #2-20:** *Lines 255-256: I suggest that the authors provide a brief summary of the findings here. What do the authors mean by "good construct validity"?*

**Response:**

We appreciate your suggestion. We have added a brief summary of our findings. By "good construct validity," we mean the SOC-13 scale's capacity to accurately measure the theoretical construct of Sense of Coherence (SOC) in the Bangla context. The results of our confirmatory factor analysis (CFA) suggest construct validity since most items load well onto their respective dimensions (See Lines 280-286). Furthermore, the model fit indices (e.g., RMSEA, CFI, TLI) are within acceptable limits, showing that the scale accurately represents the latent construct it is intended to assess. This implies that the SOC-13 scale is an appropriate tool for assessing SOC in Bangladeshi communities.

****************************

**Comment #2-21:** *Second Paragraph, Line 260: The authors mention that they considered certain pairs of items to share similar concepts during the analysis. What evidence led to this conclusion? According to Table 4, there is a weak correlation between manageability and meaningfulness in the study population. The authors paired items 4 and 10, assuming these two items share similar constructs. It would be beneficial to justify this analysis and provide evidence for and against these methods from the literature.*

*Additionally, since these items, which were modified to share covariances, belong to different domains, does this also suggest that, even though the study identified a three-factor model, there is some interchange of items between different domains? There is existing literature on modified SOC scales that supports this idea. It is well established that there is a correlation between different domains and each domain with the total SOC score, as the authors have mentioned. Have the authors considered assessing a one-factor model? Several studies have proposed a one-factor model with a good fit, which aligns with Antonovsky’s original hypothesis. Regional studies from India and Sri Lanka among young cohorts have also suggested such findings. A more in-depth discussion on this aspect would be valuable.*

**Response:**

We paired some items due to modification indices indicating potential overlap in their concepts, even though they belong to different domains. The literature suggests some overlap between SOC domains, and similar modifications to ours have been observed in other SOC research. Despite discovering a three-factor model, this suggests some item interchangeability across domains. Our data showed a better fit with the three-factor model, aligning with Antonovsky’s theory that, while interconnected, the three domains are distinct. In-depth discussion has been added (lines 300-310).

****************************

**Comment #2-22:** *Lines 271-272: The authors suggest using the SOC score as a total score. What specific findings of this study support this recommendation? Please elaborate.*

**Response:**

(Lines 310-313) We recommend using the total SOC score based on the overall good internal consistency reliability of the SOC-13 scale, as indicated by a high Cronbach’s alpha. Additionally, while we identified three dimensions (meaningfulness, manageability, and comprehensibility), the moderate to fair inter-correlations between these domains suggest they are related components of a single overarching construct. This aligns with Antonovsky’s original concept of SOC as a unified measure of resilience, supporting the use of a total score for capturing a comprehensive view of individuals' coping abilities.

****************************

**Comment #2-23:** *Lines 283-284: The statement, "Therefore, the results of this study are sufficient to demonstrate high construct validity," is unclear. Is this conclusion referring to the original SOC scale or the Bangla version? This statement needs to be rephrased for clarity.*

**Response:**

Thank you for mentioning it. The sentence was rephrased (lines 320-321) as, “*Consequently, the Bangla version of the SOC-13 scale used in this study appears to be a good measure for demonstrating high construct validity.”.*

****************************

**Comment #2-24:** *Lines 290-291: The authors claim that the sample size of the present study was inadequate and consider this a weakness. However, based on the literature on sample size determination for factor analysis, a sample size of 320 is generally considered adequate for a questionnaire with only 13 items.*

**Response:**

We have revised the sentences (lines 326-329) as, “*Further studies involving participants from different backgrounds, including various academic specialties and residential settings, may produce more robust and compelling results*”.

****************************

**Comment #2-25:** *Limitations: One of the limitations of this study could be that the authors did not investigate item and conceptual validity (conceptual equivalence). Conceptual equivalence assesses whether the constructs and domains in the original instrument are equally relevant and appropriate in the target culture. This is an important step that researchers often overlook during the adaptation of study instruments.*

**Response:**

Thank you for the valuable suggestion. According to the comment, we have added the description that we did not investigate conceptual validity in the limitation (lines 329-332).

****************************

**Comment #2-26:** *Lines 295-296: The intended meaning of this statement is unclear. Please rephrase for clarity.*

**Response:**

We rephrased this statement (lines 330-332) as follows, “*Although the translation process was skillfully conducted, equivalence across cultures in terms of language, beliefs, and context may still have scope for further examination”.*

****************************

**Comment #2-27:** *Lines 298-302: This section appears to be repeated in the conclusion section. The authors can omit this section.*

**Response:**

Thank you for suggesting it. We omitted repeated sentences (lines 334-337) as follows: “*Despite these limitations, the Bangla version of the SOC-13 will be utilized in future studies to reveal the factors associated with SOC and to obtain several suggestions on how SOC can be maintained and enhanced in Bangladesh”.* We also integrated this section into the immediately preceding paragraph.

****************************

Conclusions:

**Comment #2-28:** *The conclusion needs to be rephrased to provide a proper summary of the study findings using appropriate terms.*

**Response:**

We appreciate your valuable suggestion. The conclusion section has been revised with a proper summary using appropriate terms (lines 340-342). This is now as follows: *“The scale demonstrated good construct validity including acceptable structural validity, and good overall reliability based on internal consistency”.*
