# Supplementary material for: Psychometric properties of the Bangla version of the sense of coherence scale among university students in Bangladesh
Source: PLoS One. 2025 Apr 1;20(4):e0320896. doi: 10.1371/journal.pone.0320896 (PMC11960917; doi:10.1371/journal.pone.0320896)
Supplement: Attachment — Submitted filename: Comments_ Academic Editor.docx [file pone.0320896.s005.docx]

PONE-D-24-25645R1

Dear Dr. Yuta Hayashi

I am delighted to inform you that the review report from the reviewers has been received. Reviewer 1 accepted your manuscript, while Reviewer 2 provided comments for further development. Please address the following comments to ensure the manuscript's quality and acceptability:

Line 106- 108; “Therefore, this study aimed to translate the original English SOC-13 into Bangla and examine its construct validity and reliability based on its internal consistency among Bangladeshi university students

Comment: The sentence above implies that both construct validity and reliability were assessed using internal consistency. To improve clarity, the authors should rephrase this. Additionally, since the latter part of the manuscript specifies that both structural and convergent validity were tested, it would be more accurate to state that "validity and reliability" were assessed at this stage. Now that the methods section includes detailed descriptions of the types of validity measurements conducted, this will provide clearer information for the readers.

Line 138-140- The authors' sample size calculation and their decision to include a larger sample size are not aligned. It is rather speculative than scientifically convincing. Both smaller and larger sample sizes than calculated have limitations regarding methodological rigor and ethical considerations.

Line 207-208; convergent validity was examined using correlations with self-esteem, well-being, and psychological distress
Comment: Isn’t it discriminant validity that the authors wish to test between SOC and psychological distress?

Line 329-330- ‘Second, there was a lack of assessment of the items and cross-cultural validity’.
I assume that authors meant to say that items and concept of the questionnaire was not tested for its’ conceptual validity. Would suggest the authors to rephrase according to their intention.

Line 340-341- what about the convergent and discriminant validity? The study has shown significant positive correlation between SOC and similar construct as well as significant negative correlations between SOC and dissimilar construct. Perhaps the authors can state that in the conclusion.

Authors should discuss the study's implications in the conclusion section, considering its originality.

In addition,

- During the revision process, please use track changes or a distinct color for text modifications.
- The article's language must adhere to the journal's standards.

**Decision: Minor Revision**
